# Clinical outcomes of image-guided proton therapy for histologically confirmed stage I non-small cell lung cancer

**DOI:** 10.1186/s13014-018-1144-5

**Published:** 2018-10-11

**Authors:** Koichiro Nakajima, Hiromitsu Iwata, Hiroyuki Ogino, Yukiko Hattori, Shingo Hashimoto, Toshiyuki Toshito, Kensuke Hayashi, Kenji Akita, Fumiya Baba, Katsumi Nakamae, Jun-etsu Mizoe, Yuta Shibamoto

**Affiliations:** 1 Department of Radiation Oncology, Nagoya Proton Therapy Center, Nagoya City West Medical Center, 1-1-1 Hirate-cho, Kita-ku, Nagoya, 462-8508 Japan; 2 Department of Radiology, Nagoya City University Graduate School of Medical Sciences, 1 Kawasumi, Mizuho-cho, Mizuho-ku, Nagoya, 467-8601 Japan; 3 Department of Proton Therapy Physics, Nagoya Proton Therapy Center, 1-1-1 Hirate-cho, Kita-ku, Nagoya, 462-8508 Japan; 4 Department of Proton Therapy Technology, Nagoya Proton Therapy Center, 1-1-1 Hirate-cho, Kita-ku, Nagoya, 462-8508 Japan; 5 Department of Respiratory Medicine, Thoracic Oncology Center, Nagoya City West Medical Center, 1-1-1 Hirate-cho, Kita-ku, Nagoya, 462-8508 Japan; 6 Department of Radiation Therapy, Nagoya City West Medical Center, 1-1-1 Hirate-cho, Kita-ku, Nagoya, 462-8508 Japan; 7 Department of Thoracic Surgery, Thoracic Oncology Center, Nagoya City West Medical Center, 1-1-1 Hirate-cho, Kita-ku, Nagoya, 462-8508 Japan; 8 Osaka Heavy Ion Therapy Center, 3-1-10 Otemae, Chuo-ku, Osaka, 540-0008 Japan

**Keywords:** Stage I non-small cell lung cancer, Proton therapy, Image-guided proton therapy, Fiducial metallic marker, Respiratory gating system

## Abstract

**Background:**

Two prospective phase II trials were designed to assess the efficacy and safety of image-guided proton therapy (IGPT) for either medically inoperable or operable stage I non-small cell lung cancer (NSCLC). The present study reports the interim results of these trials.

**Methods:**

Fifty-five patients with histologically confirmed stage I NSCLC (IA in 33 patients and IB in 22 patients; inoperable in 21 patients and operable in 34 patients) who received IGPT between July 2013 and February 2017 were analyzed. The median patient age was 71 years (range: 48–88 years). IGPT with fiducial metallic marker matching was performed for suitable patients, and a respiratory gating method for motion management was used for all treatments. Peripherally located tumors were treated with 66 Gy relative biological effectiveness equivalents (Gy(RBE)) in 10 fractions (*n* = 49) and centrally located tumors were treated with 72.6 Gy(RBE) in 22 fractions (*n* = 6). Treatment associated toxicities were evaluated using Common Toxicity Criteria for Adverse Events (v.4.0).

**Results:**

Median follow-up was 35 months (range: 12–54 months) for survivors. For all patients, the 3-year overall survival, progression-free survival, and local control rates were 87% (95% confidence interval: 73–94%), 74% (58–85%), and 96% (83–99%), respectively. Fiducial marker matching was used in 39 patients (71%). Grade 2 toxicities observed were radiation pneumonitis in 5 patients (9%), rib fracture in 2 (4%), and chest wall pain in 5 (9%). There were no grade 3 or higher acute or late toxicities.

**Conclusions:**

IGPT appears to be effective and well tolerated for all patients with stage I NSCLC.

**Trial registration:**

Lung-001, 13–02-09 (9), registered 11 June 2013 and Lung-002, 13–02-10 (10), registered 11 June 2013.

## Background

For stage I non-small cell lung cancer (NSCLC), surgical resection is the current standard treatment. Stereotactic body radiotherapy (SBRT) has been adopted as an alternative treatment modality for medically inoperable patients with stage I NSCLC, and has increasingly been used for operable patients as well [1–4]. In recent years, proton therapy (PT) has been attracting attention as a new and effective treatment option. The greatest advantage of PT over photon-based SBRT is the improved dose distribution that results from the physical properties of proton beams, and in particular the Bragg peak phenomenon. Theoretically, PT enables a reduction in the unnecessary dose delivered to multiple sensitive critical organs at risk (OAR) while facilitating homogenous delivery of higher doses for the tumor [5–7].

In clinical practice, excellent outcomes of PT have been reported for stage I NSCLC [8–11]. There are, however, several outstanding technical improvements that might be made, including the use of image guidance techniques and respiratory motion management. In contrast to SBRT, the optimal irradiation method for a moving target in the body has not been well established because proton beams have more sensitive physical characteristics. Although fiducial marker implantation for image guidance and respiratory gating are widely used for SBRT, neither have been commonly used for PT. In 2013, therefore, we started phase II clinical trials of image-guided PT (IGPT) for stage I NSCLC using fiducial markers with a respiratory gating method.

In the recent several years, dozens of PT facilities have been built and their prevalence will rapidly increase over the next few years [12]. Although its efficacy has been gradually revealed by many investigators, more data is necessary to justify the recent increasing demand for PT. In Japan, medical insurance coverage of PT for stage I NSCLC is now being actively discussed and further evidence is being requested by the government. In this context, we consider that reporting the interim results of our trials is of value, especially given the promising results to date. The purpose of this study was to evaluate 3-year results from our prospective trials of IGPT for stage I NSCLC.

## Methods

### Study design

An interim analysis of ongoing prospective phase II clinical trials, named “Lung-001” and “Lung-002” and approved by the Institutional Review Board (IRB) of Nagoya City Hospital, was carried out. The IRB numbers are 13–02-09 (9) and 13–02-10 (10), respectively. The former was for medically inoperable patients, and the latter was for operable patients who refused surgery.

The sample size for these trials was calculated based on Simon’s two-stage minimax clinical phase II design [13] with a significance level of 0.05 (α = 0.05) and a power of 80% (β = 0.20). For Lung-001, the primary endpoint is incidence of grade 3 or higher radiation pneumonitis (RP) occurring within 180 days from the first date of PT. The acceptable incidence is considered to be less than 5%, and the unacceptable incidence is considered to be more than 15%. It was planned that 39 patients were to be included in Stage 1. If 3 patients or fewer experienced grade 3 or higher RP, the trial was scheduled to move on to Stage 2, with recruitment of an additional 18 patients (a total of 57 patients is required for completion of the trial). For Lung-002, the primary endpoint is 3-year overall survival (OS) rate. The acceptable and unacceptable rates are 65% and 45%, respectively. It was planned that 30 patients were to be included in Stage 1. If 17 or more survived 3 years, the trial was scheduled to move on to Stage 2, with recruitment of an additional 9 patients (a total of 39 patients is required for the trial).

Although the timing of interim analyses had not been defined at the outset, we carried out the interim analysis in response to the request from the Japanese government for up-to-date data, since the Lung-002 study has moved on to Stage 2. In the present study, only patients followed for a minimum of 12 months or until death were enrolled.

### Patient eligibility and disease staging

The inclusion criteria were as follows: 1) histologically confirmed NSCLC; 2) clinical stage IA or IB (7th edition of TNM staging of Union for International Cancer Control); 3) Eastern Cooperative Oncology Group performance status of 0–2; 4) no OARs exceeding dose constraints; 5) no previous irradiation of the treatment region; 6) no history of chemotherapy; 7) age ≥ 20 years (Lung-001), or ≥ 20 and ≤ 80 years (Lung-002), 8) forced expiratory volume 1.0 ≥ 700 mL and PaO_2_ in room air ≥60 Torr (Lung-001), or ≥ 800 mL and ≥ 65 Torr (Lung-002); and 9) written informed consent.

The exclusion criteria were: 1) pregnancy; 2) synchronous or metachronous cancer within 5 years; 3) active infectious disease; 4) other severe comorbidities, e.g., hypertension or diabetes mellitus; 5) severe psychological disorder; and 6) apparent interstitial pneumonitis or pulmonary fibrosis detectable by chest X-ray radiography. Medical inoperability was determined by multidisciplinary thoracic specialists, including a thoracic surgeon.

In all cases, staging was performed with magnetic resonance imaging (MRI) of the brain, computed tomography (CT) of the chest and upper abdomen, and ^18^F-deoxyglucose-positron emission tomography-CT (PET-CT) within 1 month before the start of PT.

### PT and treatment planning

The prescribed dose to the isocenter was 66 Gy relative biological effectiveness equivalents (Gy(RBE)) in 10 fractions for peripherally located tumors and 72.6 Gy(RBE) in 22 fractions for centrally located tumors. The biologically effective dose calculated with an α/β ratio of 10 Gy (BED_10_) was 110 and 97 Gy(RBE), respectively. All irradiation was given once a day, 5 days a week. The RBE value for our proton beams was determined to be 1.1 [14].

The treatment machines and planning system at our institution were described in detail previously [15]. Briefly, proton treatments were delivered by PROBEAT III (Hitachi, Ltd., Tokyo, Japan) and planned with VQA (Hitachi, Ltd., Tokyo, Japan). A passive scattering technique with mainly 120- to 200-MeV proton beams was used for all treatments. Two to four beam portals were used for each treatment.

**Fig. 1:**
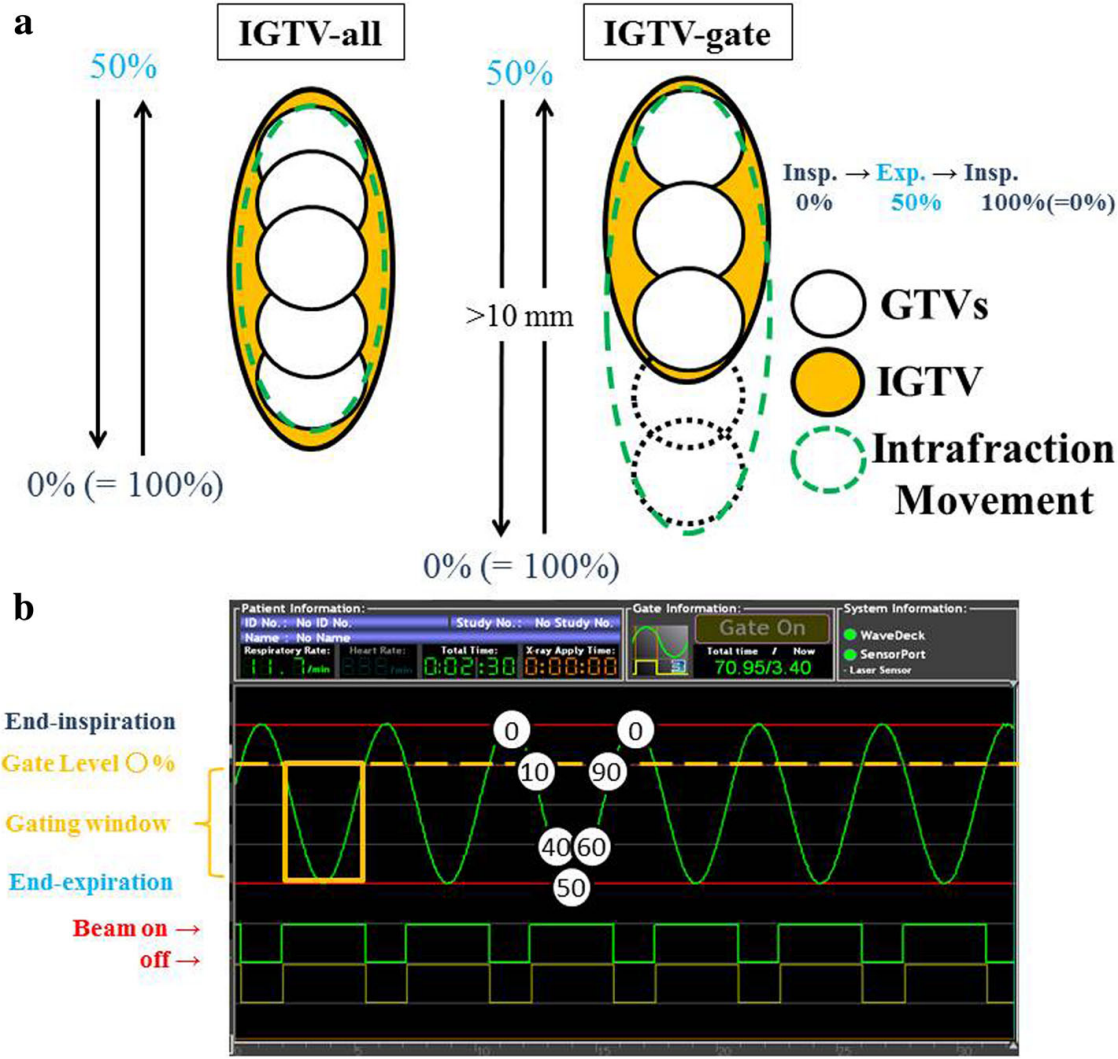
**a** IGTV volume definition. IGTV-all consisted of GTV and GTVs across all respiratory phases. IGTV-gate consisted of GTV and all GTVs within the gating window around phase 50%. The latter was only used for patients whose tumor moved more than 10 mm in any direction. **b** Schema of the respiratory gating system. Beams turned on only when the monitored respiratory phase was within the predefined gating window. The gate level was defined for each patient by reference to the amplitude of tumor movement

Prior to treatment, all patients were evaluated for their respiratory stabilities and tumor motions. In patients with a highly movable tumor, 3 or 4 fiducial markers were implanted near the tumor. The majority of markers were 1.5-mm-diameter gold markers (Olympus, Tokyo, Japan), implanted through bronchoscopy. 0.5-mm-diameter VISICOIL (RadioMed, Barlett, TN, USA) and 0.28-mm-diameter Gold Anchor (Naslund Medical Inc., Chicago, IL, USA) were percutaneously implanted in 2 and 1 patients, respectively. Patients were immobilized in the supine position using an ESFORM immobilization system (Engineering System, Nagano, Japan), and 2-mm-thick CT images were taken using a 16-row multi-detector CT (Aquilion LB; Toshiba Medical Systems, Tochigi, Japan) during the expiration phase for treatment planning. All patients underwent CT simulation with 4-dimensional (4D) CT to account for tumor motion with deformation. Patient respiratory waveforms were monitored throughout the procedures and recorded with an AZ-733 V respiratory gating system (Anzai Medical, Tokyo, Japan). After CT simulation, the 4D-CT images were reconstructed into ten respiratory phases, with end of expiration defined as phase 50% and end of inspiration as phase 0% (=100%). The tumor detected under the lung window was defined as the gross tumor volume (GTV). In addition to the GTV, GTVs from 4D-CT were contoured on CT images of each respiratory phase (GTV_0_, GTV_10_ to GTV_90_). The internal gross tumor volume (IGTV) was defined as the envelope of the GTVs. The IGTV was divided into two; IGTV-all consisted of the GTV and GTVs across all respiratory phases, and IGTV-gate consisted of the GTV and all GTVs within the gating window around phase 50%, e. g., GTV_20_ to GTV_70_ (Fig. 1a). The gating window was chosen with reference to the amplitude of marker and tumor movement. IGTV-gate was only used for patients whose tumor moved more than 10 mm in any direction. For each beam, the IGTV was expanded laterally from the perspective of the beam to encompass the setup error margin (SM) and internal motion margin (IM). The lateral margin was 6 mm for fiducial marker matching plans which included SM and only intrafractional IM. In contrast, the lateral margin for vertebral bone matching plans was 9 mm including SM and intrafractional plus interfractional IM. In addition, beam-specific distal and proximal margins to the IGTV were assigned along the beam path axis to account for uncertainties in the range of proton beams. The margins were typically 4–8 mm based on the definition of Moyers et al. [16]. To adjust the proton beam range, compensation boluses were used, and the bolus smearing margins were typically 10–18 mm [16]. The lateral and beam-specific margins were slightly adjusted to meet the dose constraints.

### Image-guidance with fiducial markers and respiratory gating irradiation

IGPT was provided for all patients. Daily patient alignments were achieved by matching fiducial markers or vertebral bones. All procedures were performed with 2D/2D matching methods using the PIAS system (Hitachi, Ltd., Tokyo, Japan) [17]. The reference digitally reconstructed radiographs (DRR) and contours for the fiducial markers were developed from the planning CT images. The procedure for fiducial marker matching was as follows: 1) patients were aligned to the isocenter using skin markers and a laser; 2) vertebral bony structure matching was performed with radiographs; 3) radiographs were taken at end-expiration and the couch was manually shifted to align the fiducial marker with the contour of the marker on the DRR, which was developed from 50% phase CT images; 4) radiographs were taken at the inspiration phase to check the correlation between the respiratory waveform and the gating window. All radiographs comprised two sets of orthogonal kilo-voltage digital radiographs.

Respiratory gating irradiation was performed by monitoring patient the waveform under free-breathing. Abdominal surface motion was used as a surrogate for tumor motion, and the beam was turned on only when the monitored respiratory phase fell within the predefined gating window (Fig. 1b).

### Treatment evaluation, follow-up, and statistical analysis

The patients were observed at 6-week intervals until 6 months after PT, and at least once every 3 months until 2 years. Thereafter, our protocol policy was to follow-up at 6-month intervals, though most patients were followed up at 3-month intervals. Regular follow-up studies included chest and upper abdominal CT scans and tumor marker examinations. MRI and PET-CT were usually performed once a year or whenever necessary. Acute and late treatment-related toxicities were assessed using the National Cancer Institute Common Toxicity Criteria for Adverse Events (v.4.0). Local recurrence was indicated by expansion of a consolidated fibrotic mass within the irradiated area on CT images, and PET-CT and biopsy were performed when recurrence was strongly suspected.

OS, local control (LC), and progression-free survival (PFS) rates were calculated using the Kaplan-Meier method from the first date of PT. Univariate associations of patient and treatment characteristics with OS, LC and PFS were examined with the log-rank test. Evaluated factors included sex, age, stage, histology, tumor site, radiation dose, position matching method and IGTV definition. A *P*-value of <.05 was considered to be significant. All statistical analyses were performed with EZR version 1.35 [18].

## Results

### Patient, tumor and treatment characteristics

**Fig. 2:**
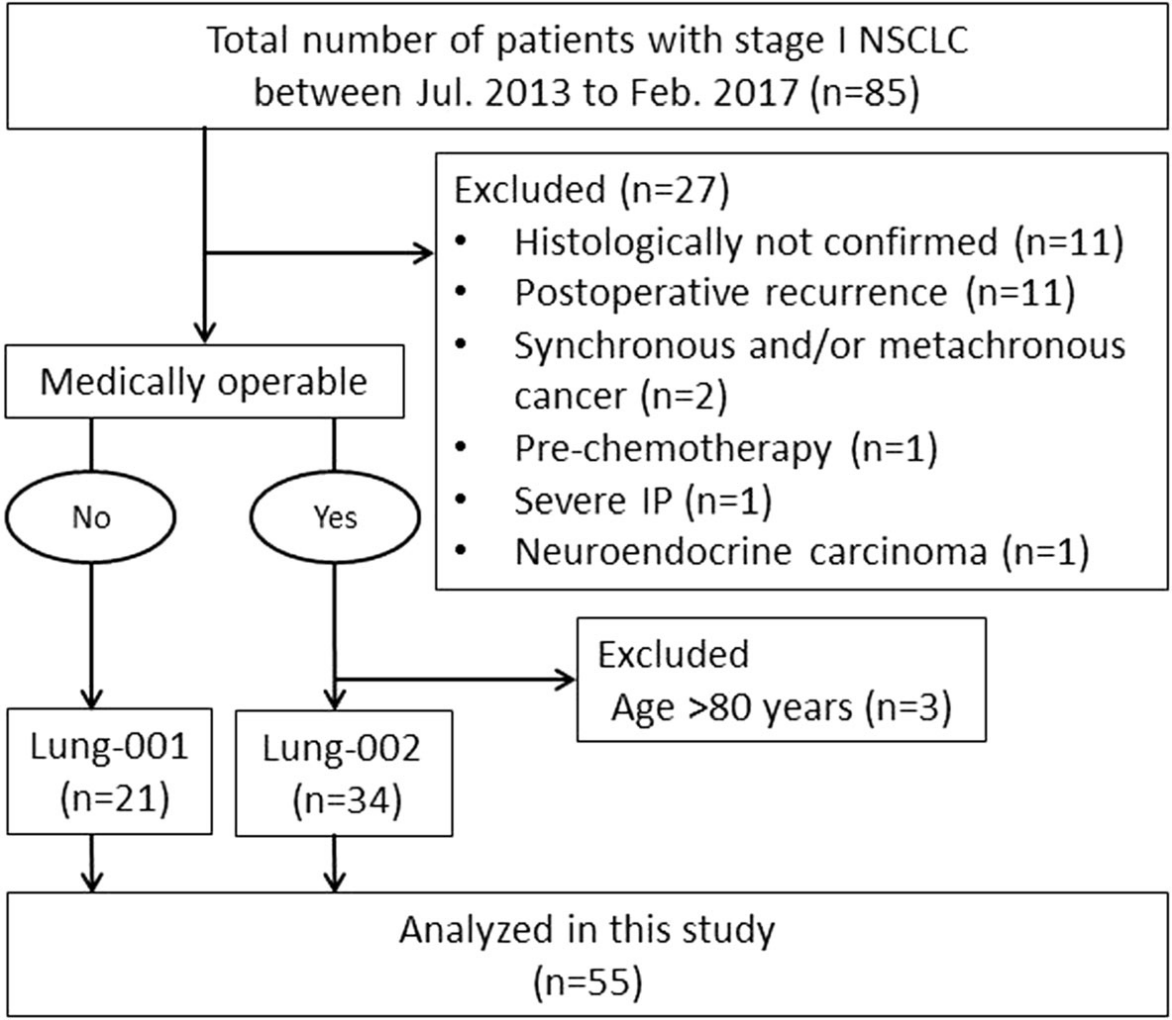
Study flow diagram. Lung-001 was for medically inoperable patients and Lung-002 was for operable patients who refused surgery

Characteristics of the patients and tumors are summarized in Table 1 and treatment characteristics are summarized in Table 2. Between July 2013 and February 2017, 85 patients clinically diagnosed with stage I NSCLC received PT; 21 inoperable patients were included in the Lung-001 study, and 34 operable patients in Lung-002. The participant study flow is depicted in Fig. 2.

**Table 1:** Patient and Tumor Characteristics

Characteristic	All	Inoperable	Operable
No. of patients	55	21	34
Age (y), median (range)	71 (48–88)	81 (65–88)	70 (48–79)
Sex			
Male	32 (58%)	15 (71%)	17 (50%)
Female	23 (42%)	6 (29%)	17 (50%)
Histology			
Adenocarcinoma	44 (80%)	13 (62%)	31 (91%)
Squamous cell carcinoma	10 (18%)	8 (38%)	2 (6%)
NSCLC	1 (2%)	0 (0%)	1 (3%)
ECOG performance status			
0	46 (84%)	12 (57%)	34 (100%)
1	7 (13%)	7 (33%)	0 (0%)
2	2 (4%)	2 (10%)	0 (0%)
Clinical stage (UICC 7th)			
Stage IA	33 (60%)	13 (62%)	20 (59%)
Stage IB	22 (40%)	8 (38%)	14 (41%)
Longest tumor diameter (mm), median (range)	27 (10–50)	27 (14–46)	27 (10–50)
Tumor location			
Center (72.6 Gy(RBE)/22 Fr)	6 (11%)	5 (24%)	1 (3%)
Periphery (66 Gy(RBE)/10 Fr)	49 (89%)	16 (76%)	33 (97%)
Tumor site			
Upper and middle lobe	41 (75%)	16 (76%)	25 (74%)
Lower lobe	14 (25%)	5 (24%)	9 (26%)
Smoking			
Yes	23 (42%)	12 (57%)	11 (32%)
No	32 (58%)	9 (43%)	23 (68%)

*Abbreviations*, *NSCLC* unclassified non-small cell lung cancer, *ECOG* Eastern Cooperative Oncology Group; UICC 7th, Union for International Cancer Control 7th edition, *Gy(RBE)* grays relative effectiveness, Fr fraction

**Table 2:** Treatment Characteristics

Characteristic	All	Inoperable	Operable
No. of patients	55	21	34
No. of portals, 2/3/4	3/41/11	0/18/3	3/23/8
Position matching method			
Fiducial marker	39 (71%)	14 (67%)	25 (74%)
Vertebral bone	16 (29%)	7 (33%)	9 (26%)
IGTV definition			
IGTV-all	48 (87%)	18 (86%)	30 (88%)
IGTV-gate	7 (13%)	3 (14%)	4 (13%)
GTV (cc), median (range)	11 (1.1–53)	12 (1.7–38)	10 (1.1–53)
IGTV (cc), median (range)	17 (1.8–80)	22 (2.8–53)	15 (1.8–80)
DVH parameters			
IGTV V95% (%), median (range)	100 (97–100)	100 (99–100)	100 (97–100)
IGTV D95% (%), median (range)	100 (97–100)	99 (98–100)	100 (97–100)
Lung-GTV V5 (%), median (range)	14 (4.3–28)	14 (4.3–28)	13 (5.2–25)
Lung-GTV V20 (%), median (range)	9.3 (3.0–21)	9.6 (3.0–21)	8.7 (3.7–18)

*Abbreviations, IGTV* internal gross tumor volume, *GTV* gross tumor volume, DVH dose-volume histogram. V95% = volume receiving > 95% of the prescribed dose, D95% = minimum doses covering 95% of the volume, V5, 20 = volume receiving dose > 5/20 Gy(RBE)

### Survival and local control

At the time of analysis, 48 patients were alive and 7 patients were dead. Six of the dead patients were in the inoperable group, including 3 deaths from lung cancer and 3 deaths from other causes, while 1 patient in the operable group died of another cause. The median follow-up was 31 months (range: 9–54 months) for all patients and 35 months (range: 12–54 months) for the patients who remained alive. Local recurrence occurred in 2 patients: one with T1a tumor in the inoperable group at 20 months after PT, diagnosed without biopsy; and another with T2a tumor in the operable group at 16 months after PT, diagnosed with biopsy. Regional lymph node recurrence was observed in 2 patients and distant metastasis was observed in 5. The patterns of failure are shown in Table 3.

**Table 3:** Patterns of Failure

Pattern	All(n = 55)	Inoperable(n = 21)	Operable(n = 34)
Local	2 (4%)	1 (5%)	1 (3%)
LN	2 (4%)	2 (10%)	0 (0%)
Distant	5 (9%)	2 (10%)	3 (9%)

*Abbreviations*, *LN* regional lymph nodes. Distant metastases included ipsilateral (*n* = 1), contralateral (n = 1), bilateral (n = 2), and pleural dissemination (n = 1)

**Fig. 3:**
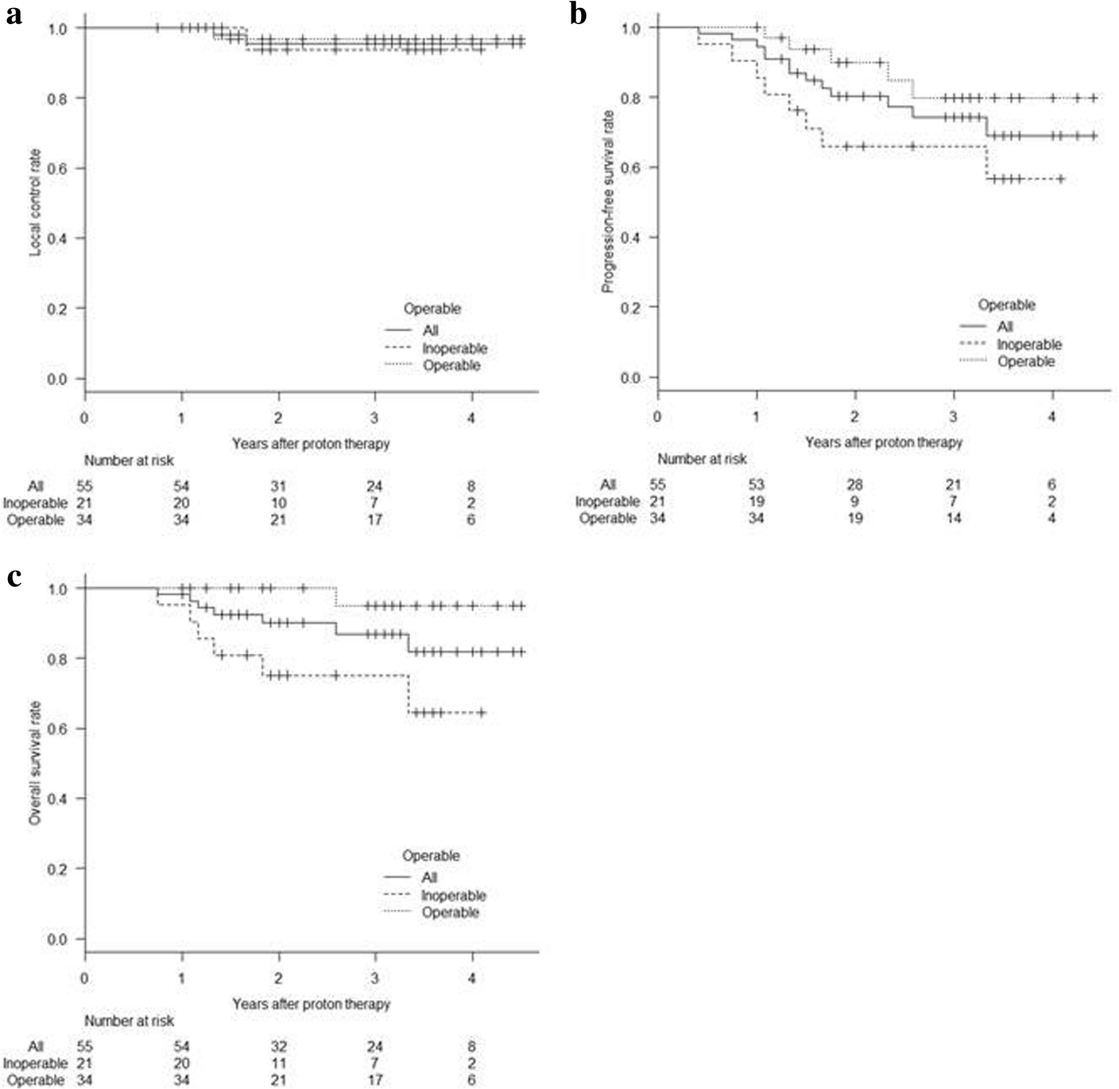
Kaplan-Meier curves for local control **a**, progression-free survival (**b**) and overall survival (**c**) for all patients, medically inoperable and operable groups

For all patients, the 3-year OS, PFS, and LC rates were 87% (95% confidence interval [CI]: 73–94%), 74% (58–85%), and 96% (83–99%), respectively (Fig. 3). For inoperable patients (*n* = 21), the 3-year OS, PFS and LC were 75% (95% CI: 50–89%), 66% (42–82%), and 94% (63–99%), respectively. For operable patients (*n* = 34), the 3-year OS, PFS and LC were 95% (70–99%), 80% (95% CI: 57–91%), and 97% (79–100%), respectively. In univariate analysis, tumor site (3-year OS: 95% for upper/middle lobe tumors and 75% for lower lobe tumors; *P* = .021), age (3-year OS: 100% for ≤71 years and 77% for > 71 years; *P* = .018) and operability (*P* = .0066) were associated with OS. Tumor site (3-year PFS: 86% for upper/middle lobe tumors and 39% for lower lobe tumors; *P* = .0020), histology (3-year PFS: 82% for adenocarcinomas and 43% for squamous cell carcinomas; *P* = .040), and age (3-year PFS: 90% for ≤71 years and 63% for > 71 years; *P* = .037) were associated with PFS. No factors were correlated with LC.

### Toxicities

Table 4 summarizes toxicities associated with treatments. No grade 3 or higher toxicities were observed. Five patients (9%) developed grade 2 symptomatic RP at 2 to 7 months from the beginning of PT. All patients received steroids and their symptoms improved. Grade 2 rib fracture and chest wall pain caused by soft tissue inflammation were observed in 2 (4%) and 5 patients (9%), respectively. No symptomatic dermatitis was observed. In addition, there were no other grade 2 toxicities noted during either acute or late observation periods.

**Table 4:** Toxicities

Toxicity	All (*n* = 55)	Inoperable (*n* = 21)	Operable (*n* = 34)
Grade 2			
Radiation pneumonitis	5 (9%)	2 (10%)	3 (9%)
Rib fracture	2 (4%)	0 (0%)	2 (6%)
Chest wall pain	5 (9%)	1 (5%)	4 (12%)
Dermatitis	0 (0%)	0 (0%)	0 (0%)

The toxicities were evaluated according to the Common Terminology Criteria for Adverse Events version 4.0. There were no grade 3 or greater toxicities

## Discussion

This study reported clinical outcomes of IGPT using fiducial markers and respiratory gating for operable and inoperable histologically-confirmed stage I NSCLC. IGPT provided 3-year OS and LC rates of 87% and 96%, respectively, with low incidence of toxicities. A systematic review of 9 studies on particle beam therapy (including 6 PT and 5 carbon-ion radiotherapy (CIRT) studies) and 72 SBRT studies has previously been carried out. The 3-year OS, PFS, and LC rates for particle beam therapy were 70, 64, and 88%, respectively, compared to 59, 51, and 86%, respectively for SBRT [19]. No statistically significant survival benefit from particle beam therapy over SBRT was observed after adjusting for potential confounding variables. Our results obtained using IGPT compare favorably with the results of the aforementioned analysis. In addition, we observed no grade 3 toxicities. Although the incidence of severe toxicity was low in both PT or SBRT groups, grade 3 or higher RP was less frequent (PT vs. SBRT, 0.9% vs. 3.4%; *P* < .001) whereas chest wall toxicity was more frequent (1.9% vs. 0.9%; *P* = .03) in the PT group [19]. Furthermore, any-grade rib fractures were observed more frequently in PT (13% vs. 3.2%; P < .001). Chest wall toxicity and rib fracture in PT could be problems of critical importance because they compromise patient quality-of-life (QOL). To clarify the advantages of PT, randomized trials should be conducted, but this is practically difficult. Chest wall toxicities and rib fractures after PT may be reduced by employing more portals, and the physical characteristics of PT should be advantageous in reducing toxicities so that it may be possible to further escalate doses to the tumors [5–7, 20].

The best strategy to employ when using PT for stage I NSCLC remains open to discussion, particularly with respect to the IGPT method and optimal fractionation schedule. We employed fiducial marker matching for suitable patients. Fiducial marker or tumor matching are widely used in SBRT for lung or liver tumors [21, 22], but they have not been commonly used in PT, probably because changes in surrounding anatomical structures (especially the bone) affect the PT dose distribution. Reports from a CIRT group suggested, however, that fiducial marker or tumor matching was better than bony structure matching [23, 24]. We adopted a respiratory gating method, which has been performed in SBRT and other PT facilities, though most PT facilities have uniformly defined IGTV as enveloped GTVs from all respiratory phases with or without 4D-CT. As is well documented in a report about SBRT [25], the IGTV might be unnecessarily large in some cases. In our strategy, choosing IGTV-all or IGTV-gate depending on the amplitude of tumor movement can help to reduce unnecessary doses to critical OAR for specific patients. We selected suitable patients who could possibly benefit from these irradiation methods because PT has several uncertainties and should be operated as personalized treatment [26]. This might have resulted in the absence of severe toxicities and the low rate of local recurrence in the present study. Moreover, this policy might have yielded the results that no factors were associated with LC or grade 2 toxicities (data not shown).

Current PT involves various emerging technologies, including pencil-beam or spot scanning, real-time imaging and gating system, and in-room or cone beam CT among others [27–29]. The current study suggests, however, that in terms of both efficacy and safety, PT is suitable for adoption as a standard treatment option for stage I NSCLC even without such technologies. To confirm the therapeutic benefit of PT, more cases and longer follow-up will be needed. Furthermore, the optimal fractionation regimens with possible dose escalation should be investigated further. Using fewer fraction numbers, especially for central tumors, would increase patient throughput, and this approach may be adopted in the next study. To determine an optimal fractionation regimen, both physical high-precision technology and reasonable biological knowledge are necessary [30]. Over time, the unique biological effects of proton beams, compared to photon beams, have been gradually clarified [14, 31–33]. We will evaluate more effective fractionation regimens focused on PT from both physical and biological aspects.

This study has several limitations. This is an interim analysis of ongoing prospective phase II clinical trials. As such, the sample size was relatively small and the follow-up period relatively short. In addition, to perform IGPT with our method, we have carried out physical analysis of dose distribution, margin definition and respiratory motion effects; the results could not be reported in this article and will be reported in future. Another limitation of the present study is the lack of patient QOL and cost-effectiveness assessment. We have collected the QOL data, but as the data on patients with short follow-up are not yet obtained, we will include the data in the final reports.

## Conclusions

Our results indicate that PT is an effective and safe treatment option for stage I NSCLC. Patient-specific optimal irradiation methods may contribute to better clinical outcomes. It is important to select suitable treatment strategies for each patient because patients and tumors are heterogeneous and PT has uncertain sensitive characteristics. Further studies are needed to demonstrate the true value of PT.

## Abbreviations

CIRT: Carbon-ion radiotherapy
DRR: Digitally reconstructed radiographs
GTV: Gross tumor volume
Gy(RBE): Gray relative biological effectiveness equivalents
IGPT: Image-guided proton therapy
IGTV: Internal gross tumor volume
IM: Internal motion margin
OAR: Organs at risk
PT: Proton therapy
RP: Radiation pneumonitis
SBRT: Stereotactic body radiotherapy
SM: Setup error margin
